# Decadal drought deaccelerated the increasing trend of annual net primary production in tropical or subtropical forests in southern China

**DOI:** 10.1038/srep28640

**Published:** 2016-06-30

**Authors:** Wantong Wang, Jinxia Wang, Xingzhao Liu, Guoyi Zhou, Junhua Yan

**Affiliations:** 1 College of Tourism, Henan Normal University, Xinxiang 453007, China; 2 South China Botanical Garden, Chinese Academy of Sciences, Guangzhou 510650, China; 3 College of Landscape, Fujian Agriculture and Forestry University, Fuzhou 350002, China

## Abstract

Previous investigations have identified that the effects of climate change on net primary production (NPP) of global forests have varied both spatially and temporally, and that warming has increased the NPP for many forests. However, other factors, such as available soil water for plant growth, could limit these incremental responses to warming. In our investigation we have quantified the responses of NPP of tropical or subtropical forests in southern China to warming and drought stress over the past three decades (1981 to 2012) using data from five forest research stations and satellite measurements. NPP, mean annual temperature (MAT) and annual days without rainfall showed an increase of 0.076 g C m^−2^ a^−2^ (standardized), 0.057 °C a^−1^ (standardized) and 0.067 d a^−1^ (standardized) during the study period, respectively. However, incremental NPP was deaccelerated at a rate of approximately 20.8% per decade. This deacceleration was primarily caused by a decrease in available soil water which resulted from warming (mainly occurring in winter and autumn) and the changes in rainfall pattern. The result indicates that intensifying drought stress would limit future increases of forest NPP in southern China.

## Influence of climate change on annual NPP

Net primary production (NPP) is an important measure of terrestrial ecosystem functioning^1^. NPP represents the ability of terrestrial plants to fix atmospheric CO_2_, and it is also a key flux of the global carbon cycle^2^. Climate warming directly influences terrestrial NPP by changing the carbon uptake capacity of leaves; it also indirectly affects NPP by changing soil water availability^3^ and by prolonging the growing season^4^.

Temperature, water and radiation interact to impose complex and variable limitations on NPP in different parts of the world^5^. Many studies have revealed temporal changes in terrestrial ecosystem NPP in response to continental or global scale climate change^5^,^6^,^7^,^8^,^9^. In the temperature-limited regions at high latitudes, such as North America^10^ and Northern China^11^,^12^, warming extended the plant growing season by promoting earlier plant growth and their NPP. Nevertheless, in predominantly radiation-limited regions over the middle and low latitudes, such as the Amazon basin^13^,^14^,^15^, an increase in solar radiation, owing to declining cloud cover, is the most likely explanation for increased NPP of tropical forests.

## The influence of climate change on TEBF ecosystems and carbon cycling

**Figure 1:**
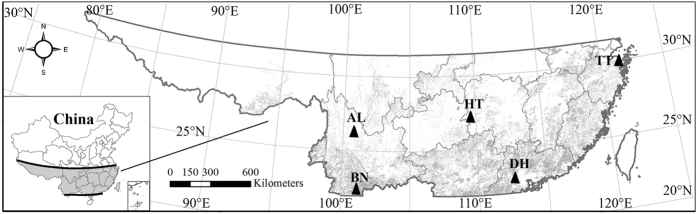
Locations of the five field stations in China’s tropical and subtropical evergreen broadleaved forests(Map created using ARCGIS 10.1 software by first author, URL: http://www.esri.com). The five field stations are TT-Tiantongshan, DH-Dinghushan, HT-Huitong, BN-Xishuangbanna, and AL-Ailaoshan.

China’s tropical and subtropical evergreen broadleaved forests (TEBF) are located in the region between 20°N to 31°N and 101°E to 122°E, an area which covers more than 26% of China’s land surface (Fig. 1). Over the past several decades, dramatic climatic changes have occurred in this region. Mean annual temperature (MAT) has increased at a rate of 0.2 °C decade^−1^ from 1952 to 2011 (this value is higher than the 0.17 °C decade^−1^ on a global scale recorded between 1948 and 2010)^6^, whilst mean annual precipitation (MAP) has remained unchanged^16^. Furthermore, rainfall patterns have shifted towards more severe storms. As a result, the soils have become drier and extreme hydrological events (e.g. droughts and floods) have become more frequent^17^. Overall, such rapid warming and change in rainfall patterns has led to an increase in drought stress, thereby reducing tree diameter growth^18^, enhancing tree mortality^19^,^20^, and decreasing the biomass carbon sink^21^. Zhou *et al*., investigating TEBF biome changes based on the observations from established permanent plots over the past four decades, reported that TEBF ecosystems have undergone a transition from cohorts with few large individuals to ones with a larger number of smaller individuals. They suggested that these subtropical forests were under threat due to their lack of resilience to these changes^22^. The reorganization of these biomes was the result of regional warming and associated soil drying^16^, findings which were consistent with results from drought experiments in the Amazon forest^23^.

However, few studies have examined the impact of climate change on TEBF in southern China. There are still some urgent questions which need to be answered, such as (1) how will the TEBF ecosystems’ NPP changes under such a long-term warming and the consequences of drought stress; and (2) what are the main limiting climate factors affecting NPP in this region, and what impact do they have? In this study, we compiled and analyzed NPP data based on the AVHRR GloPEM NPP data^24^ (1981–2000) and the MOD17A3 NPP data^25^ (2001–2012), and analyzed the corresponding climatic variables and soil moisture across five forest field research stations in the TEBF (Fig. 1 and Table 1) from 1981 to 2012. Pearson correlation analyses were performed to determine the climatic factors that caused significant changes in the NPP. Finally, we synthesized the latest findings on the effect of drought stress on TEBF ecosystems and carbon cycling.

## Results

### Annual or decadal changes in climate variables

Table 2 shows the change in climate variables for the study region from 1981 to 2012. Overall, there was a significant increase at the rate of 0.057 °C a^−1^ (standardized) in MAT and of 0.067 d a^−1^ (standardized) in annual days without rainfall. For mean annual relative humidity (MARH), annual days with small rainfall, and soil water content in the top 50 cm, there was a significant decrease at the rate of 0.062% a^−1^ (standardized), 0.048 d a^−1^ (standardized), and 0.063 mm a^−1^ (standardized), respectively. For MAP, no significant change was identified.

**Figure 2:**
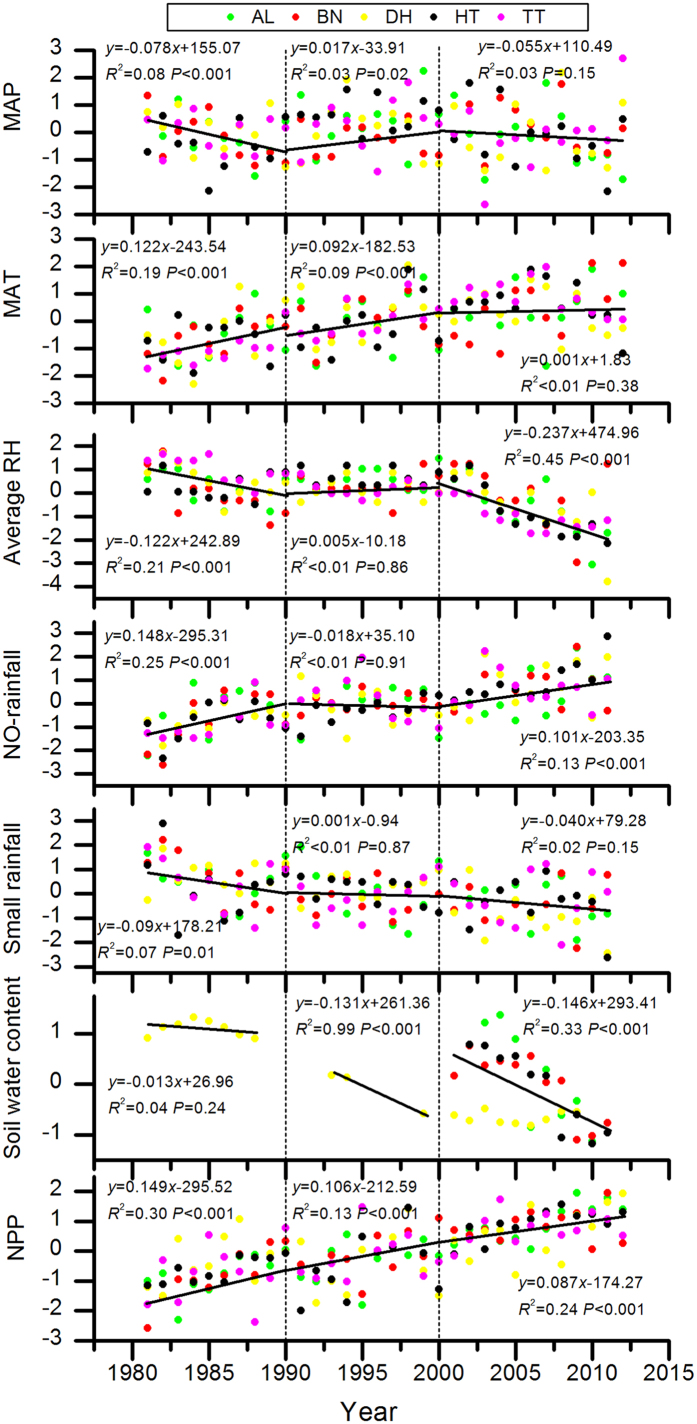
Decadal trends of the standardized data representing climate variability and net primary production in the tropical or subtropical evergreen broadleaved forests over the past three decades. Soil water content (mm) was obtained from plots located at four stations (AL, BN, DH, HT).

However, changes observed in different decades were different from annual changes for the climate variables and soil water content over the study period (Fig. 2). Firstly, in the different decades, the changes for the climate variables and soil water content were different. Results for MAP, for example, showed a decrease at the rate of 0.078 mm a^−1^ (standardized) in the first decade (from 1981 to 1990), an increase at the rate of 0.017 mm a^−1^ (standardized) in the second decade (from 1990 to 2000), and a decrease at the rate of 0.055 mm a^−1^ (standardized) in the third decade (from 2001 to 2012). Compared with this, as already stated, there were no significant trends for MAP over the study period (Table 2). For MARH, and annual days with small rainfall, apart from significant downtrends being identified from 1981 to 2012, similar characteristic changes to those identified for MAP in the three decades were identified. The significance of climate variable change was also identified to be different. For example, a significant uptrend was identified for MAT in the first two decades, but in the third decade no significant change was identified. For annual days without rainfall, a significant uptrend was shown in the first decade and the third decade, yet no significant change was identified in the second decade. Overall, significant changes for the climate variables mainly occurred in the first and third decades, and a significant downtrend for soil water content mainly occurred in the recent two decades.

### Monthly or seasonal variations in trends of climate variables

**Figure 3:**
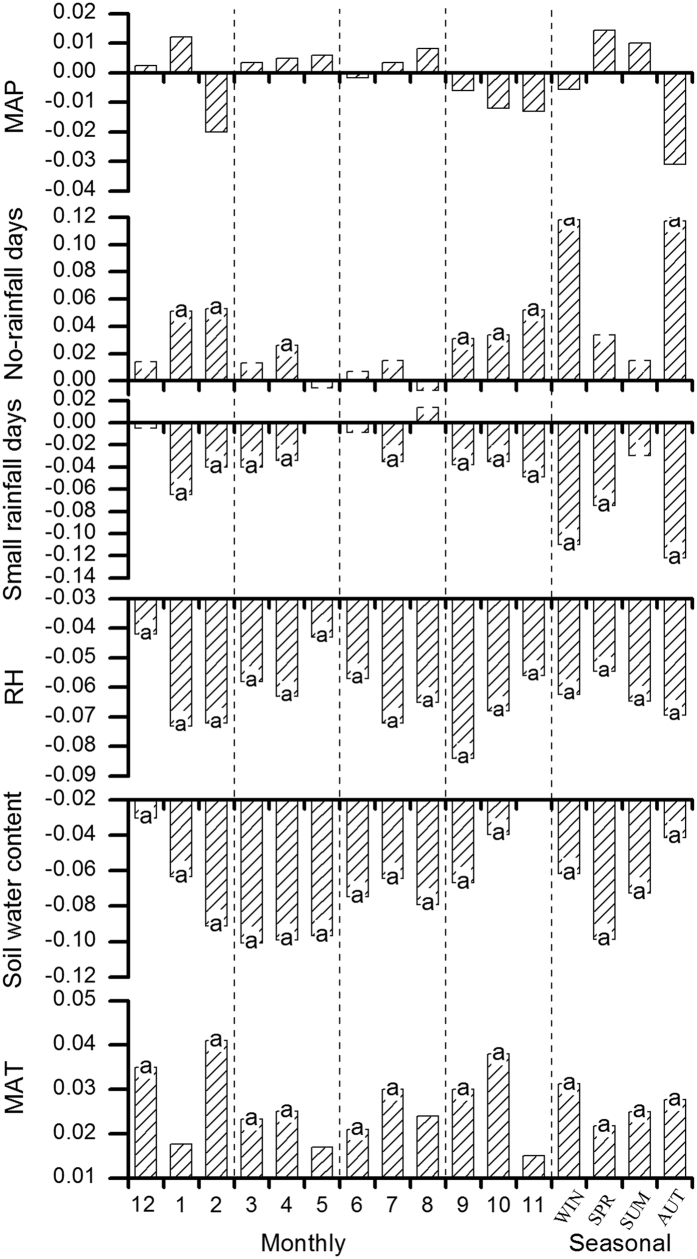
Variations in change rate of standardized data representing climate variables or net primary production at the monthly or seasonal scales in tropical or subtropical evergreen broadleaved forests from 1981 to 2012. Soil water content (mm) was obtained from the plots located at four stations (AL, BN, DH, HT). The slope obtained from a least square linear regression was used to estimate the rates (the letter “a” above histogram bar signifies the coefficient by statistical significance test (*P *< 0.05).

Climate variables showed large variations within months or seasons (Fig. 3). Overall, MAT increased the fastest in winter, followed by autumn, summer, and then spring, which led to a reduction in inter-seasonal differences in temperature. At the monthly scale, the fastest increase in MAT occurred in February and October. MAP increased in the spring and summer and decreased in the autumn and winter, but these differences were not significant. This led to an increase in the uneven distribution of seasonal precipitation: the wet-season became wetter and the dry-season became drier. Annual days with small rainfall and annual days without rainfall showed opposite trends in which the former decreased more quickly in the autumn (mainly occurring in November) and winter (mainly occurring in January and February) than in spring and summer, while the latter increased faster in the same season and months than the former. This resulted in extended dry periods and severe droughts. MARH rates exhibited smaller differences between seasons but had similar characteristics as displayed by annual days with small rainfall. Soil water content decreased faster in the spring and summer than in the winter and autumn, but the intensity of the decrease was contrary to that in MARH. This implied that the drought in the soil was delayed by two seasons compared to the atmosphere.

### Annual NPP trends

As shown in Table 2 and Fig. 2, annual NPP increased at a rate of 0.076 g C m^−2^ a^−2^ (standardized) from 1981 to 2012, but with variations of the rate of increase between the three decades; 0.149 g C m^−2^ a^−1^ in the 1980s, 0.106 g C m^−2^ a^−1^ in the 1990s and 0.087 g C m^−2^ a^−1^ for 2000–2012 (all values were standardized). Therefore, the increase of NPP was deaccelerated by 20.8% per decade over the study period.

### Relationships between NPP trend and climate variables

Over the study period (1981 to 2012) there were strong correlations between annual NPP and climate variables, except for MAP (Table 3). The results showed that annual NPP was strongly positively correlated with annual days without rainfall (*r *= 0.510, *P *< 0.001) and MAT (*r *= 0.508, *P *< 0.001); whereas it was significantly negatively correlated with MARH (*r *= −0.491, *P *< 0.001), annual days with small rainfall (*r *= −0.352, *P *< 0.001), and the soil water content of the top 50 cm (*r *= −0.320, *P *= 0.025).

For the seasonal patterns, the correlation between annual NPP and annual days without rainfall was strongly positive in autumn (*r *= 0.330, *P *< 0.001) and winter (*r *= 0.266, *P *< 0.001), while for annual days with small rainfall there was a negative correction (*r *= −0.285, *P *< 0.001; *r *= −0.232, *P *=  0.004, respectively). Annual NPP was strongly positively correlated with MAT in the autumn (*r *= 0.423, *P *< 0.001), winter (*r *= 0.350, *P *< 0.001), summer (*r *= 0.324, *P *< 0.001), and spring (*r *= 0.242, *P *= 0.002), and was strongly negatively correlated with MARH in the same four seasons (all *P *< 0.001). Overall, NPP was influenced in the winter and autumn by changes in rainfall patterns (annual days without rainfall and annual days with small rainfall), while MAT and MARH had an equal influence all year round.

As shown in Table 4, annual NPP and climate variables (annual days without rainfall, MAT and MARH) were significantly correlated, as determined by stepwise multi-regression analysis. This result was consistent with the above findings. It was possible to explain the annual NPP variance that accounted for 39.9%. Standardized coefficients represented the contribution of the independent variables when explaining the dependent variable change. In this study, they represented the influence of each climate variable on annual NPP. Therefore, annual days without rainfall were the most important factor for annual NPP, followed by MAT and then MARH. Within the total effects, 33.5% was due to MAT and 46% was due to changes in rainfall patterns (annual days without rainfall).

## Discussion

TEBFs are located in China’s humid region where adequate water conditions ensure the sustained growth of forest vegetation. Our results showed an increasing trend for NPP at the rate of 0.076 g C m^−2^ a^−1^ from 1981–2012 (about 7.9% from 1982 to 1999), which was greater than the global rate (about 6% from 1982 to 1999)^8^. However, within each of the three decades, the increasing rate of NPP showed a decreasing trend of 20.8% per decade. The dynamic change in NPP implied that forest ecosystems in this region were negatively impacted by climate change. Recent reports have confirmed this effect, and suggested that TEBF ecosystems experienced some significant changes, such as reorganization of biomes^16^, reduction of tree diameter growth^18^, and enhanced tree mortality^20^,^22^, all of which were due to drought stress. Severe drought conditions can not only have a negative impact on tree growth in a moist tropical forest, it can also lead to a large volume of carbon emissions due to forest fires and tree mortality^23^. On the other hand, our findings show that the threats caused by climate change have yet to overcome the resilience of TEBF ecosystems^22^, as shown by the continued increase of NPP over the long-term period.

During 1981–2012, in the study region, the anisomerous change in climate variables between seasonal and decadal timescales has resulted in stronger droughts, especially soil droughts in the growing season (spring and summer), but this drought trend has mainly occurred in the most recent decade due to a lack of water (as shown in Fig. 2, MAP, MARH, and soil water content significantly decreasing between 2000–2012). Recently, NASA data has shown that there was a declining trend in plant growth worldwide due to droughts which has caused global plant productivity to only increase by 1% from 2000 to 2009^7^.

Climate, at both regional and global scales, has a significant effect on ecosystem processes^26^. Temperature, water and other factors interact to impose complex and varying limitations on NPP in different parts of the world^27^. When there is sufficient water, increases in temperatures and solar radiation can promote plant photosynthesis to increase NPP, whereas drought stress can reduce photosynthesis and enhance autotrophic respiration, which together reduce NPP. Among all the climate variables we studied, annual days without rainfall and annual days with small rainfall control the precipitation pattern; their changes resulted in more drought events by gathering precipitation (more no-rainfall days) and increasing evapotranspiration (more sun radiation) to enhance the loss of water^28^. On the other hand, more radiation can promote plant photosynthesis to increase NPP when soil water is not significantly limiting. For MAT, it is confirmed that a warming climate can lengthen the growing season^4^,^29^; furthermore, under drought stress, warming indirectly dampens photosynthesis as the plant tries to reduce water loss by reducing stomata conductance that causes a decline in NPP. Therefore, the interaction between temperature and precipitation conditions and the related changes in the climate caused the observed variations of NPP at our study sites.

At high latitude or in semi-arid regions, rainfall is rare and unchanged, and temperature exerts the dominant control on plant growth by lengthening the growing seasons^29^; however, in low latitudes or humid regions, water availability has the dominant control on plant growth^7^. Our results also showed that annual days without rainfall and MAT exerted the most dominant influences on NPP based on the correlation and multi-regression analysis in the study region. During the 1980s when soil water was not limiting, MARH and soil water content had less variation and warm winters enhanced plant growth in this region, and then during the 1990s, under weak drought stress, soil water content decreased. This change resulted in a reduction of the vegetation’s photosynthesis and the increasing rate of annual NPP declined by 28.9% than in the 1980s, although there being an increasing trend due to MAT. From 2000 to 2012, severe drought stress (MARH and soil water content decreased quickly) resulted in a continuous reduction in the increasing rate of NPP. Overall, the increasing rate of NPP was deaccelerated due to drought stress that was caused by anisomerous changes in seasonal warming and rainfall patterns in the region.

Overall, the anisomerous change in the rate of climate variables between months and seasons has resulted in warmer winters and stronger droughts in this region, especially during the growth season (spring and summer). It can also be predicted that as warming increases and rainfall patterns change in the future, stress due to drought conditions will continue to intensify and limit any future increase in forest NPP. This will therefore destabilize the forest carbon balance and weaken the terrestrial carbon sink in this region.

## Materials and Methods

### Site description

As the regional climax vegetation, China’s TEBF dominates the directions for natural succession and artificial restoration of all degraded forest ecosystems in the region^30^. A subtropical monsoon climate prevails in the study region due to its proximity to the South China Sea, the general air circulation, and the existence of the Qin-Tibet plateau. This climate has an MAP of 1300–2000 mm; 80% of precipitation occurs in the wet season (April to September) and 20% occurs in the dry season (October to March). MAT and MARH are 15.0 ~ 21.5 °C and 78%, respectively^30^.

Across this region, since the 1970s, the Chinese Academy of Sciences and the Ministry of Science and Technology of the People’s Republic of China have deployed a series of field research stations. We selected five of these field research stations (Tiantongshan TT, Dinghushan DH, Huitong HT, Xishuangbanna BN, and Ailaoshan AL) (Fig. 1 and Table 1) to investigate the influence of climate change on TEBF ecosystems’ NPP. It was confirmed that, up until the study date, the chosen ecosystems had not experienced artificial management or natural catastrophic events (landslides, typhoons, forest fires, etc.), and that no traces of such events can be found. In addition, for the whole plot, the soil profiles are well preserved. Thus, the five stations selected provided sufficient information about the response of the TEBF ecosystems to climate change under natural conditions.

### Climate variables and soil moisture monitoring

Long-term meteorological data from field stations close to the study sites were downloaded from the database of the Chinese National Meteorological Information Center/China Meteorological Administration (NMIC/CMA). For this study, five climate variables (MAP, MAT, MARH, annual days without rainfall, and annual days with small rainfall) were selected.

Soil moisture was measured using both a neutron probe and gravimetric sampling in eight plots located at four stations (DH, HT, BN, and AL). All results were converted into volumetric water content (%) of the respective soil layers after being combined with soil bulk density data. Moisture located in the top 50 cm of the soil was selected as the soil water factor.

For comparison among different sites, all of the compiled data were standardized using Equation 1:
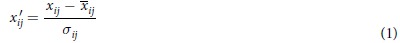
where, *x′*_*ij*_ is the standardized data that corresponds to the original data *x*_*ij*_, dimensionless; *i* represents the station; *j* represents the census year; 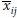 represents the means of the measurement values in the respective durations for the station *i*; and *σ*_*ij*_ represents the SD of measurement values in the respective durations for station *i*.

### NPP data processing

An increasingly important role in identifying changes in terrestrial NPP is being played by satellite sensors. The annual NPP dataset used in this study was generated from the GloPEM (1981–2000) and MOD17A3 (2000–2012) data. The GloPEM dataset is derived from Advanced Very High Resolution Radiometer (AVHRR) images at an 8 km resolution, as obtained from the AVHRR Path finder Project. As a part of NASAs Earth observatory System (EOS) program, MOD17 MODIS has produced the first satellite derived dataset to recorded vegetation productivity at a global scale. Here we used the MOD17A3 annual NPP data at a 1 km resolution and the annual NPP data from GloPEM. Due to both NPP data sources containing the same year (2000), a relative correction method was used to remove the differences. First, to keep the same resolution and better accuracy, the MOD17A3 data (1 km resolution) were resampled into an 8 km resolution using the bilinear interpolation method. A number of group samples were then extracted in the same position nearby the study area from the two processed NPP data sets (2000) which were used to analyze correlations. The regression equation (*y *= 0.278*x* + 590.8, *R*^2^* *= 0.56) was used to adjust the MOD17A3 data. Then, as the final step, the NPP values (2001–2012) were extracted from the adjusted MOD17A3 data, and the NPP values (1981–2000) were directly extracted from the GloPEM NPP data for each of the five stations. All the data processing was performed using ARCGIS (version 10.1, ESRI) software.

### Statistical analysis

A simple linear regression model was used to regress the trends of standardized data representing NPP and climate variables for the five sites. Person correlation analysis was performed to identify the relationship between the standardized data representing annual NPP and climatic variables; the contributions of the standardized data representing climatic variables on NPP were calculated with stepwise multi-regression analysis. Statistical analysis and data processing were conducted using SPSS (version 19.0, SPSS Inc.) software.

## Additional Information

**How to cite this article**: Wang, W. *et al*. Decadal drought deaccelerated the increasing trend of annual net primary production in tropical or subtropical forests in southern China. *Sci. Rep.* **6**, 28640; doi: 10.1038/srep28640 (2016).

## Figures and Tables

**Table 1:** Description of the five sites.

**Site name**	**Forest type**	**Dominant species**	**Forest age(year)**	**Location**	**Altitude(m)**	**MAT(°C)**	**MAP(mm)**
AL	Subtropical evergreen broadleaved forest	*Lithocarpus xylocarpus, Castanopsis rufescens*	>250	101°01′E, 24°32′N	2488	11.3	1982
BN	Tropical seasonal rain forest	*Pometia pinnata, Terminalia myriocarpa, Gironniera subaequalis*	>100	101°12′1″E, 21°57′ 40″N	750	21.5	1557
DH	Monsoon evergreen broadleaved forest	*Castanopsis chinensis, Schima superba, Michelia odora*	>400	112°32′22″E, 23°10′11″N	220	21.0	1956
HT	Subtropical evergreen broadleaved forest	*Castanopsis hystrix, Cyclobalanopsis*	>250	109°36′E, 26°50′N	350	16.5	1300
TT	Subtropical evergreen broadleaved forest	*Castanopsis fargesii*	>200	121°47′12″E, 29°48′29″N	196	17.1	1408

**Table 2:** Trends in the standardized data representing annual climate variability and net primary production in tropical or subtropical evergreen broadleaved forests from 1981 to 2012.

	***b***	**SE**	**R**^**2**^	***P***	***n***
MAP~	−0.001	0.008	<0.001	0.884	160
MAT↑	0.057	0.007	0.286	<0.001	160
MARH↓	−0.062	0.007	0.314	<0.001	160
Top 50 soil water content↓	−0.063	1.153	0.460	<0.001	49
Annual days without rainfall↑	0.067	0.007	0.373	<0.001	160
Annual days with small rainfall↓	−0.048	0.008	0.193	<0.001	160
NPP↑	0.076	0.522	0.511	<0.001	160

Note: ↑significant uptrend; ↓significant downtrend; ~no significant directional changes.

**Table 3:** Pearson’s correlation coefficients between the standardized data representing climate variability and annual net primary production in tropical or subtropical evergreen broadleaved forests from 1981to 2012.

**Climate variables**	*r*					*n*
	**WIN**	**SPR**	**SUM**	**AUT**	**Annual**	
MAP	−0.031	0.090	−0.052	−0.232	−0.120	160
MAT	0.350**	0.242*	0.324**	0.423**	0.508**	160
MARH	−0.474**	−0.281**	−0.435**	−0.516**	−0.491**	160
Top 50 soil water content	−0.193	−0.237	−0.321*	−0.220	−0.320*	49
Annual days without rainfall	0.266**	0.110	0.121	0.330**	0.510**	160
Annual days with small rainfall	−0.232*	−0.172*	−0.064	−0.285**	−0.352**	160

^*^P < 0.05, ^**^P < 0.001.

**Table 4:** Multiple-regression models of the standardized data representing annual net primary production for the tropical or subtropical evergreen broadleaved forests in China from 1981 to 2012.

**Regression equation**	**Standardized Coefficients**		***P***	***R***^**2**^	**SE**	***n***
y = 0.354x1+0.256x2−0.157x3 − 0.031			0.012	0.399	0.765	160
	*x*_1_	0.355	0.001			
	*x*_2_	0.258	0.003			
	*x*_3_	0.158	0.012			
	*x*_4_	0.063	0.364			
	*x*_5_	0.081	0.591			
	*x*_6_	0.022	0.818			

Dependent variables: *y*: NPP, Independent variables: *x*_1_: annual days without rainfall; *x*_2_: .MAT; *x*_3_: MARH; *x*_4_: MAP; *x*_5_: soil water content; *x*_6_: annual days with small rainfall.
